# Iron-loaded carbon black prepared *via* chemical vapor deposition as an efficient peroxydisulfate activator for the removal of rhodamine B from water

**DOI:** 10.1039/d3ra04566h

**Published:** 2023-09-04

**Authors:** Harez R. Ahmed, Kosar Hikmat Hama Aziz, Nian N. M. Agha, Fryad S. Mustafa, Steven John Hinder

**Affiliations:** a Department of Chemistry, College of Science, University of Sulaimani Qlyasan Street Sulaimani City 46001 Kurdistan Region Iraq kosar.hamaaziz@univsul.edu.iq harez.ahmed@univsul.edu.iq; b College of Science, Department of Medical Laboratory Science, Komar University of Science and Technology Sulaimani 46001 Iraq; c Department of Medical Laboratory of Science, College of Health Sciences, University of Human Development Sulaimaniyah Iraq; d Department of Mechanical Engineering Sciences, Faculty of Engineering and Physical Sciences, University of Surrey Guildford Surrey GU2 7XH UK

## Abstract

The excessive use of organic pollutants like organic dyes, which enter the water environment, has led to a significant environmental problem. Finding an efficient method to degrade these pollutants is urgent due to their detrimental effects on aquatic organisms and human health. Carbon-based catalysts are emerging as highly promising and efficient alternatives to metal catalysts in Fenton-like systems. They serve as persulfate activators, effectively eliminating recalcitrant organic pollutants from wastewater. In this study, iron-loaded carbon black (Fe-CB) was synthesized from tire waste using chemical vapor deposition (CVD). Fe-CB exhibited high efficiency as an activator of peroxydisulfate (PDS), facilitating the effective degradation and mineralization of rhodamine B (RhB) in water. A batch experiment and series characterization were conducted to study the morphology, composition, stability, and catalytic activity of Fe-CB in a Fenton-like system. The results showed that, at circumneutral pH, the degradation and mineralization efficiency of 20 mg L^−1^ RhB reached 92% and 48% respectively within 60 minutes. Fe-CB exhibited excellent reusability and low metal leaching over five cycles while maintaining almost the same efficiency. The degradation kinetics of RhB was found to follow a pseudo-first-order model. Scavenging tests revealed that the dominant role was played by sulfate (SO_4_^−^˙) and superoxide (O_2_^−^˙) radicals, whereas hydroxyl radicals (OH˙) and singlet oxygen (^1^O_2_) played a minor role in the degradation process. This study elucidates the detailed mechanism of PDS activation by Fe-CB, resulting in the generation of reactive oxygen species. It highlights the effectiveness of Fe-CB/PDS in a Fenton-like system for the treatment of water polluted with organic dye contaminants. The research provides valuable insights into the potential application of carbon black derived from tire waste for environmental remediation.

## Abstract 2

Fe-CB was synthesized *via* CVD from tire wastes and used for the degradation and mineralization of RhB by persulfate based advanced oxidation processes.

## Graphical abstract

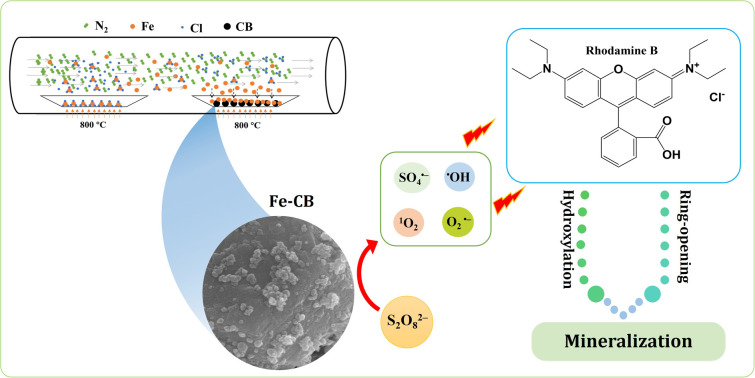

## 1. Introduction

Organic compounds have been widely used in industrial activity, agriculture, and food processes. Industry produces significant amounts of wastewater containing both conventional and emerging pollutants. Emerging pollutants are harmful chemicals not yet regulated by national or international laws.^1,2^ Organic pollutants cannot be fully removed by the common treatment methods of traditional sewage treatment plants owing to their antibacterial properties and recalcitrance, thus causing the contamination of the aquatic environment.^3^ Water pollution by emerging organic pollutants is a serious environmental problem that affects the quality and availability of freshwater resources.^4^ Organic pollutants include a wide range of substances that are derived from natural or anthropogenic sources, such as organic dyes, pesticides, pharmaceutical residues, oil spills, and agricultural runoff.^5,6^ Among them, synthetic dyes employed in textile manufacturing are considered to be highly detrimental, non-biodegradable, carcinogenic, and resistant to environmental degradation.^7^ Rhodamine B is an organic dye that is extensively used in the textile industry for dyeing and printing of fabrics. It is known to be a potential environmental pollutant due to its persistence and toxicity, which can have adverse effects on aquatic life and human health.^8,9^ Therefore, it is urgent to find an efficient method to degrade organic pollutants to manage and control the discharge of these pollutants into water bodies. Conventional wastewater treatment plants are unable to effectively remove emerging pollutants present in wastewater feed streams.^10,11^ Physicochemical methods such as adsorption by activated carbon^12,13^ and carbon xerogel decorated with multiwalled carbon nanotubes^14–16^ are effective and widely used techniques for the removal of organic dyes from aqueous solution. However, these techniques do not decompose the pollutants, but only transfer them from the aqueous phase to the solid adsorbent. This can lead to the risk of environmental contamination, as the sludge produced may contain harmful pollutants. In comparison to alternative treatment methods such as biological treatment,^17^ membrane separation,^18^ and adsorption,^19^ advanced oxidation processes (AOPs) are a promising and efficient water treatment technology for removing emerging organic pollutants *via* producing reactive oxygen species (ROS).^20,21^ The ROS group includes hydroxyl, sulfate, superoxide radicals, and singlet oxygen. ROS may be produced *via* the activation of peroxymonosulfate (PMS) and peroxydisulfate (PDS) utilizing methods such as heat, UV radiation, metal ions, metal oxides, and so on.^22–24^ Fenton-like oxidation based on the activation of persulfate (PMS or PDS) has garnered considerable attention as a promising method for the degradation and mineralization of recalcitrant organic pollutants, owing to its exceptional features, including excellent degradation capacity, ease of operation, cost-effectiveness, and non-toxicity.^25,26^ The use of iron-loaded catalysts for the catalytic activation of persulfate is deemed efficient, as the conventional Fenton process has some limitations such as a limited pH range and high sludge yield.^27^ Iron-loaded carbon catalyst has gained considerable attention in heterogeneous Fenton-like oxidation in recent years.^28,29^ The use of iron has the advantages of superior catalytic properties compared to other metals with catalytic nature such as copper and cobalt, and it is also cost-effective, easily recoverable, and has low toxicity.^29^ Secondly, the redox performance of Fe-loaded carbon black can be controlled by regulating various Fe/C molecules.^30^ Thirdly, the iron-loaded carbon itself has a strong pollutant removal ability by adsorption, which can further enhance the effectiveness of heterogeneous Fenton-like processes.^31^ The raw materials for the production of iron-loaded carbon black are widely available, including tire wastes agricultural residues, sewage sludge, municipal organic solid waste, and industrial wastes.^32^

Chemical vapor deposition (CVD) has emerged as a widely utilized material processing technology in recent times, as it enables the formation of thin films on a heated substrate through a chemical reaction of gas-phase precursors. This method offers a distinct advantage in terms of its effectiveness and simplicity, as it relies on chemical reactions that allow for the adjustment of deposition rates and the production of high-quality products with exceptional conformality.^33^ Thermal CVD employs higher temperatures to facilitate chemical reactions, resulting in slower nucleation rates and lower coverage. Atmospheric pressure CVD, however, operates at lower pressure and temperature, allowing for easier vaporization of precursors and improved film quality due to kinetically controlled reactions dominated by surface reaction rates.^34^

**Scheme 1:**
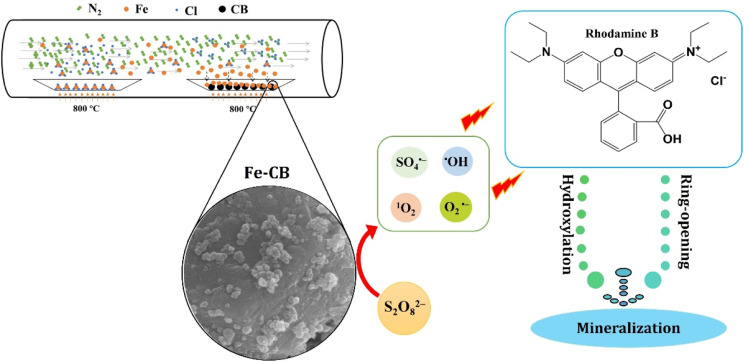
The processes of preparation of Fe-CB *via* chemical vapor deposition as a PDS activator to generate ROS and removal of RhB from water.

This study aimed to prepare Fe-CB from tire waste *via* chemical vapor deposition and utilize it as a Fenton-like catalyst to activate PDS for the removal of RhB from water (Scheme 1). The experimental parameters were optimized, and the synthesized catalyst was characterized. Furthermore, the mechanism of PDS activation by Fe-CB was proposed and discussed, focusing on reactive species analysis. These findings offer valuable technical support and theoretical evidence for tire waste recycling and the efficient removal of organic dye pollutants.

## 2. Material and methods

### 2.1. Materials

Carbon Black (CB) was produced by collecting and processing raw material obtained from a waste tire recycling factory in the Kurdistan region of Iraq into a black powder. The tire waste was collected and cut into pieces, then gradually heated to 360 °C in a furnace. The process resulted in the conversion of 45% of the waste into fuel oil, 20% into iron, and 3–4% into black powder. The black powder was then sieved to a particle size of 120 mesh and utilized as the feed material in pyrolysis experiments. These experiments were conducted in a tubular electric furnace, with a diameter of 38 mm and a length of 300 mm. For each run, 5 g of the sample was placed in a ceramic boat within the furnace, and nitrogen gas was used as a carrier gas with a constant flow rate of 3 mL s^−1^ to maintain an inert atmosphere. The reactor was heated at a rate of 10 °C min^−1^ from room temperature to 800 °C for 2 hours and finally cooled down to room temperature. Rhodamine B (RhB, ≥99%) was purchased from Acros Organics, while sodium peroxydisulfate (PDS, ≥99%), sodium hydroxide (NaOH, ≥99%), nitrobenzene (NB, ≥99%), and sulfuric acid (H_2_SO_4_, ≥98%) were obtained from Biochem Chemopharma. Ferric nitrate nonahydrate (Fe (NO_3_)_3_·9H_2_O, ≥99%), ethanol absolute, *p*-Benzoquinone (*p*-BQ, ≥98%), and l-histidine (l-H, ≥99%) were purchased from Riedel-de haen AG. All chemicals were of analytical grade and used without purification, while reagents were prepared using deionized water. The raw material (carbon black) can be cheaply provided from a waste tire recycling factory, as it is a residual waste of about 3–4% after the conversion of 45% of the waste into fuel oil, 20% into iron, and 3–4% into black powder. The commercial price of PDS is about 4.36 US$ per m^3^.^35^ The cost of electricity for preparing catalysts depends on the electricity rate in each country.

### 2.2. Preparation and characterization of Fe-CB catalyst

In the laboratory, the iron-loaded carbon black was synthesized through co-pyrolysis of tire wastes and iron(iii) chloride using chemical vapor deposition under N_2_ pyrolysis at a temperature of 800 °C. The precursor and the CVD reactor were placed in furnaces for the synthesis reaction. The furnaces were constructed from quartz glass to withstand the high temperatures of 800 °C. A vacuum was utilized to increase the volatility of the precursor, and a cold trap made of stainless steel was positioned within a water bath to condense any unreacted precursor and safeguard the vacuum pump. Anhydrous FeCl_3_, purchased from Merck Co. Ltd, was employed as the precursor with a melting point of 306 °C. This compound undergoes sublimation at 172 °C before melting, resulting in the decomposition of the dimeric vapor into solid ferrous chloride and chlorine, and the decomposition of solid ferric chloride into solid ferrous chloride and chlorine. At a temperature below 500 K, the primary iron-containing vapor species in equilibrium with FeCl_3_(cr) is the dimer Fe_2_Cl_6_.^36^ Nitrogen was utilized as the carrier gas with a flow rate ranging from 1 to 3 mL s^−1^. The evaporation temperature was established at 180 °C. An inadequate temperature would result in the precursor not evaporating, while an excessive temperature would necessitate maintaining the CVD reactor at 800 °C to decompose the precursor. After the deposition process, nitrogen gas was infused into the reactor for 4 hours at 800 °C to convert the metal nitride into metal and deposit it onto carbon black (CB). Additionally, the Fe-CB was thoroughly washed with dilute HCl ten times finally with D. W. till pH reached neutral and subjected to drying in an oven at 80 °C for a period of 12 hours.

The morphology and surface elemental composition of synthesized catalysts were characterized by using scanning field emission scanning electron microscopy (FESEM) in combination with energy-dispersive X-ray spectroscopy (FESEM-EDS) (SEM, S-400N, Hitachi). The X-ray powder diffraction (XRD) analysis was conducted using X-ray diffraction (XRD), (PHILIPS, XPert-MPD). The XRD system employed a 40 kV/30 mA X-ray generator and a two-dimensional semiconductor X-ray detector to obtain diffraction peaks *via* continuous 2*θ* scanning within a range of 10–80°, at a 2° min^−1^ scanning rate. The obtained data were subsequently calibrated with the annealed silicon standard specimen in 2*θ* calibration, and the X-ray target source used for the analysis was Cu-Kα with a wavelength of 1.5406 Å. The surface functional groups of the synthesized catalyst were examined using Fourier Transform Infrared Spectroscopy (FTIR) with a PerkinElmer Spectrum One instrument from the USA, with a resolution of 2 cm^−1^ and a wavenumber range of 4000–400 cm^−1^. The Brunauer–Emmett–Teller (BET, Belsorp mini II from Microtrac Bel Corp., Japan) was used to evaluate the specific surface area using nitrogen adsorption–desorption and pore diameter distribution studies. X-ray photoelectron spectroscopy (Thermo Scientific™ K-Alpha™ X-ray Photoelectron Spectrometer) was used to examine the elemental valences. The magnetic properties of the synthesized catalyst were analyzed using a vibrating sample magnetometer (MDKB).

### 2.3. Mechanistic analysis of catalyst formation

In this study, FeCl_3_ was used as the precursor, which has a relatively low melting point and boils at approximately 306 °C. The vapor generated from the precursor consists of the dimer Fe_2_Cl_6_, which increasingly dissociates into its monomer form at higher temperatures. This dissociation occurs concurrently with the reversible decomposition of the precursor, as shown in eqn (1), producing FeCl_2_ and Cl_2_. Nitrogen was used as the carrier gas with a flow rate ranging from 1 to 3 mL s^−1^ to ensure the transportation of FeCl_2_. At elevated temperatures, FeCl_2_ decomposes to form FeN_2_, which further decomposes at 400 °C as illustrated in eqn (2).

To thermally decompose the precursor and reduce the metal nitride into metal, the CVD reactor was maintained at 800 °C for 4 hours, depositing it onto carbon black, as shown in eqn (3). The resulting sample at this stage was labeled as Fe-CB0 and was not subjected to any further treatment. However, the iron leaching test results for Fe-CB0 showed a level of 18 mg L^−1^, which exceeds the maximum contaminant level set by the Environmental Protection Agency of 3 mg L^−1^. To address this issue, the catalyst was washed several times with dilute HCl to regenerate any FeN_2_ and FeCl_2_ bonded to carbon black to FeCl_3_ and remove them from the catalytic solution. The catalyst was then washed with distilled water (D. W.) until a neutral pH was achieved. After washing, the iron leaching test was repeated, resulting in a reduced leaching level of 12 mg L^−1^. Subsequently, the catalyst was dried at 80 °C. In cases where any physical bonds of FeCl_3_ and FeN_2_ with CB remained during the washing process, magnet force was employed to separate those chemically bonded with Fe-CB, which was magnetically adaptable. Any remaining unbonded species were separated and not bonded to the magnet. Following these treatments, the catalyst was labeled as Fe-CB, and the iron leaching test was performed again, revealing a significant reduction to a level of 0.6 mg L^−1^, as shown in Fig. 1.1
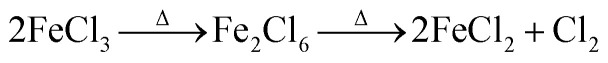
2
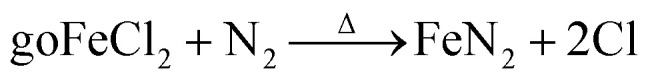
3
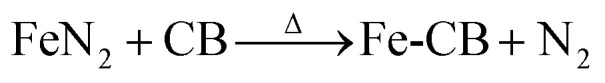


**Fig. 1:**
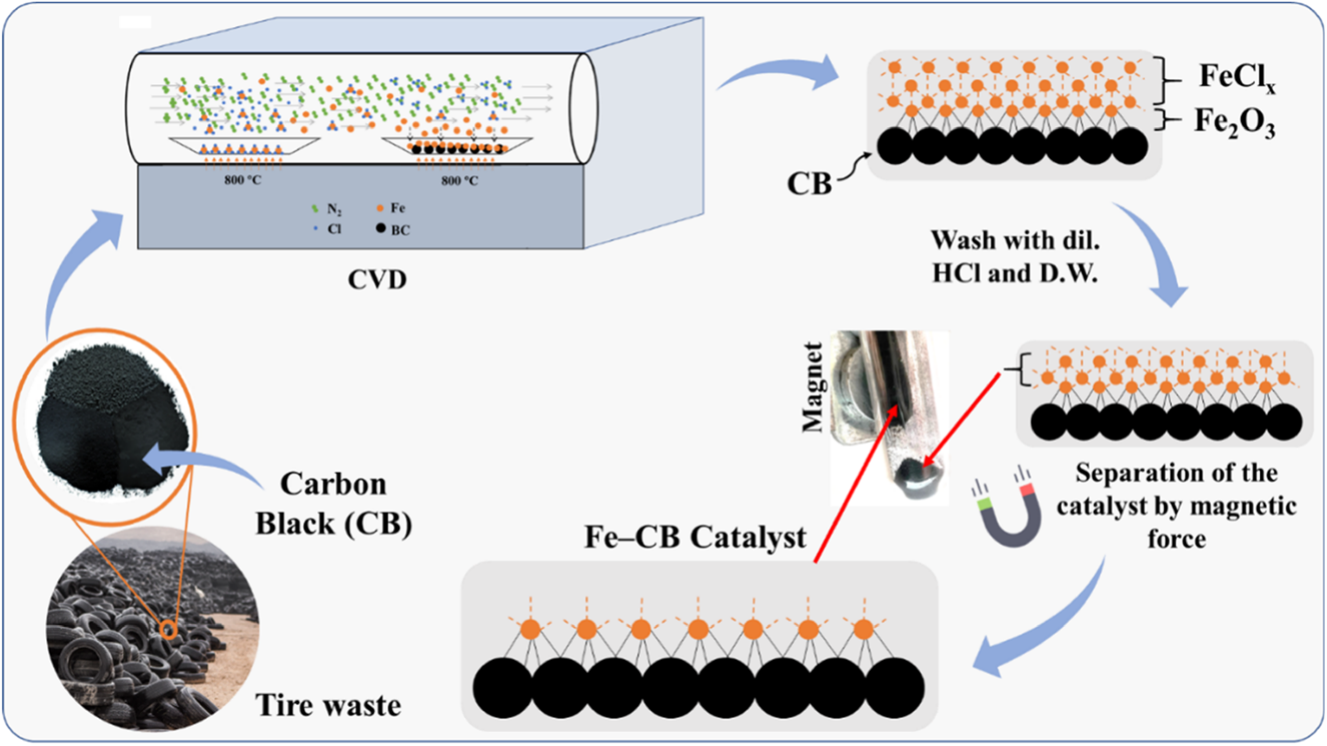
A mechanistic investigation of Fe-CB formation *via* CVD, and its separation from physical vapor deposition utilizing magnetic force.

The formation mechanism of Fe-CB from hydrocarbon moieties of iron complex involves the catalytic activity of elemental iron, which is formed by the decomposition of the iron complex at elevated temperatures in the chemically inert and highly reducing environment of the chemical vapor deposition (CVD) chamber. This decomposition produces various iron species, including Fe_1−*x*_O, magnetite (Fe_3_O_4_), ferric oxide (Fe_2_O_3_), iron carbide (Fe_3_C), and elemental carbon. Iron carbide is unstable in a carbon-rich environment at high temperatures and decomposes into Fe and C. The formation of elemental iron catalyzes the formation of Fe-CB. The magnetite in the composite is likely formed during the cooling phase from the deposition temperature, due to the disproportionation of Fe_1−*x*_O into Fe and Fe_3_O_4_ at temperatures below 576 °C. The final product consists of a composite of Fe, Fe_3_O_4_, Fe_2_O_3_, and C, with the carbon taking the form of Fe-CB under optimal CVD conditions. The deposition of Fe_2_O_3_ during the process makes it highly sensitive to the presence of oxygen; even minute leaks in the CVD system can affect the process's reproducibility. Moreover, any change in the precursor's decomposition pathways under CVD conditions significantly affects the deposit's composition.

### 2.4. Experiments and procedure

The degradation experiments were conducted in a 100 mL beaker containing 20 mg L^−1^ RhB solution at a temperature of 25 °C. To determine whether the removal of RhB is due to catalytic degradation or adsorption reaction, an adsorption experiment was conducted using only the Fe-CB catalyst, without adding PDS, in the RhB solution under identical experimental conditions. The degradation procedure involved the addition of Fe-CB to the solution, followed by the addition of PDS. At specified intervals, a 1 mL sample was withdrawn from the solution and the catalyst was filtered out using a 0.22 μm organic filter. The reaction was then quenched by adding an adequate amount of methanol, and the resulting samples were analyzed at 554 nm using a UV-vis spectrophotometer. In the recycling experiment, the Fe-CB that had been utilized in the previous degradation experiment was separated from the reaction mixture using a magnet, and then washed to prepare it for use in the subsequent degradation experiment. The leached concentrations of iron ions were measured using an inductively coupled plasma optical emission spectrometer. For the scavenger experiments, EtOH, NB, l-histidine, and *p*-BQ were employed as quenchers. All other reaction conditions were identical to those described above, except for the addition of quenchers. These scavenger experiments enabled us to identify the primary reactive oxygen species (ROS) responsible for the degradation of RhB in Fe-CB/PDS Fenton-like systems. The data are reported as mean values, and all procedures were repeated at least twice (*n* ≥ 2).

### 2.5. Analysis

The initial and residual concentrations of RhB were measured by UV-visible spectrophotometer at 554 nm (TU-1800S). The Inductively Coupled Plasma Optical Emission Spectrometer (ICP-OES), specifically the PerkinElmer Optima 2100 DV model, USA, was used to quantify the concentrations of leached Fe ions during and after the degradation process. The mineralization efficiency measured by total organic carbon (TOC) removal of RhB was determined by utilizing the TOC analyzer TOC-VCPH Shimadzu.

## 3. Results and discussion

### 3.1. Characterization of Fe-CB catalyst

**Fig. 2:**
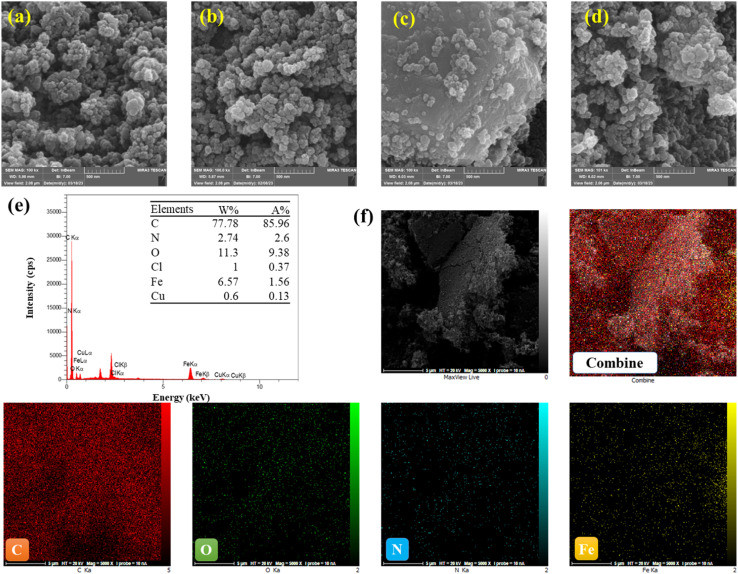
FESEM images of (a) pristine carbon black, (b) Fe-CB, and (c) modified Fe-CB, (d) used Fe-CB (e) shows the EDS spectrum of Fe-CB, with the inset displaying the pie chart of elemental compositions, (f) exhibits elemental mapping of C, O, Fe, and N.

FESEM and EDS mapping were used to display the surface morphologies and particle sizes of the Fe-loaded catalyst. The results are presented in Fig. 2a, which indicates that the product primarily consists of monodisperse particles with diameters varying from 40 to 55 nm. Furthermore, Fig. 2b presents an SEM image of Fe-CB before treatment, highlighting the prevalence of nanoparticles with diameters around 35 nm. Moreover, FESEM imaging of the Fe-CB nanoparticles (also shown in Fig. 2b) indicates the presence of nearly monodisperse microspheres with diameters of roughly 24 nm. The surfaces of the Fe_2_O_3_ microspheres seem to be rough.^37^Fig. 2c displays a FESEM image of the modified Fe-CB resulting from treatment with dilute HCl and magnet force. The resulting Fe-CB nanoparticles feature smooth and bright surfaces, indicating a core–shell structure resulting from the coupling of CB onto the Fe_2_O_3_ surface, with a particle diameter ranging from 36 to 39 nm. Additionally, Fig. 2d presents a FESEM image of the used Fe-CB nanoparticles, indicating the adhesion of numerous tiny Fe_2_O_3_ nanoparticles with diameters of 34–35 nm to the carbon black layer. Fig. 2e, along with EDS mapping profiles (Fig. 2f), reveal a homogeneous distribution of Fe, N, O, and C atoms throughout the carbon black. The presence of multiple C, O, and Fe, and small N components, uniformly distributed on the catalyst surface, indicates the successful formation of the Fe-CB composite. Notably, the EDS mapping of Fe-CB after treatment exhibits a higher degree of homogeneity compared to Fe-CB before treatment, using Fe-CB, and CB.

**Fig. 3:**
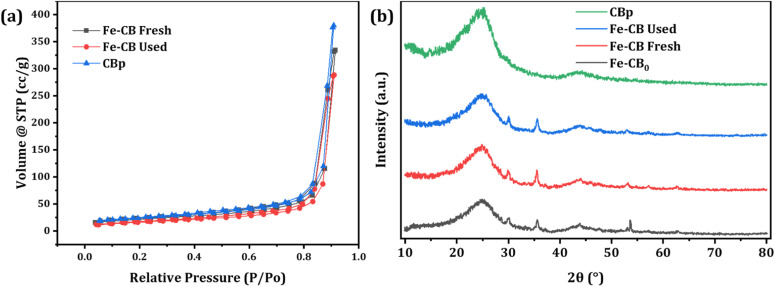
(a) N_2_ adsorption–desorption isotherm curves of the synthesized catalysts. (b) XRD spectrum of the synthesized catalysts.

Nitrogen sorption isotherms were conducted to assess the BET-specific surface area (BET-SSA) distribution of the prepared catalysts, as shown in Fig. 3a. According to the IUPAC classification system, the sorption isotherms of CB, Fe-CB fresh, and Fe-CB used exhibited a type II isotherm characterized by a long and narrow loop at a relative pressure (*P*/*P*_0_) range of 0.1 to 1.0. This isotherm is commonly observed in non-porous or macroporous adsorbents that exhibit unlimited monolayer-multilayer adsorption. Initially, the adsorption volume rapidly increased at low relative pressures due to the contact of the adsorbate molecules with the higher energetic section, followed by interaction with the less energetic section. The formation of monolayer adsorbed molecules was complete, followed by multilayer formation corresponding to the “sharp knee” of the isotherms. Finally, as the relative pressure approached unity, there was a sudden rise indicating the bulk condensation of the adsorbate gas to liquid.^38^ The BET-specific surface area for CBp after heat treatment was reported to be 74.463 m^2^ g^−1^, and the pore volume was 0.6149 cm^3^ g^−1^.^39^ The effects of chemical vapor deposition (CVD) on Fe-CB fresh and Fe-CB used can be observed as their BET-SSA values (68.108 m^2^ g^−1^ and 55.918 m^2^ g^−1^, respectively) are lower than that of CBp. This indicates successful impregnation of Fe_2_O_3_ into the pores. The decrease in specific surface area and pore volume is advantageous for the adsorption and degradation of organic contaminants, as it exposes more active sites, resulting in enhanced catalytic efficiency.

X-ray diffraction (XRD) was used to analyze the crystal structures and elemental composition of the synthesized Fe-CB catalysts, as shown in Fig. 3. The investigation aimed to determine the structural parameters of CBp, Fe-CB, and Fe-CB after-treatment processes. Fig. 3b illustrates the XRD pattern of the raw CB, which exhibits a broad peak width and a pronounced baseline. Notably, the 002 peaks of crystalline graphite, which should be observed at a diffraction angle (2*θ*) of 26.56°, are detected near 25.1°, indicating a micro-crystalline structure that differs from that of graphite. The XRD analysis successfully identified the crystal structures and composition of Fe-CB (Fig. 3b) and determined the structural parameters of CB, Fe-CB, and used Fe-CB after treatments.^40,41^ XRD was used to analyze the catalyst before and after treatment. The resulting XRD patterns of Fe-CB showed reflection peaks at diffraction angles (2*θ*) of 30.10°, 35.32°, 42.86°, 56.96°, and 62.44°. These peaks are consistent with the characteristic reflections of the rhombohedral structure of Fe_2_O_3_ (JCPDS no. 39–1346), which suggests the formation of Fe_2_O_3_ microspheres. The peaks at 2*θ* = 30.10° and 35.32° correspond to the (104) planes, while the peak at 2*θ* = 42.86° corresponds to the (110) plane. The absence of any other peaks confirms the presence of only pure α-Fe_2_O_3_ (JCPDS no. 39-1346).^42^ XRD analysis of other oxides, including Fe_3_O_4_, α-Fe_2_O_3_, and γ-Fe_2_O_3_ samples showed no secondary peaks in the spectra and similar peak positions at 2*θ*. The chemical vapor deposition (CVD) method employed nitrogen as a protective agent against the oxidation of divalent iron salts. Under CVD conditions of 800 °C for 4 hours, the Fe_3_O_4_ nanoparticles underwent a phase transition and transformed into Fe_2_O_3_, which indicates high sample purity.^43^ The treatment process had no significant impact on the formation of Fe_2_O_3_ nanoparticles, and the peaks at a diffraction angle (2*θ*) of 52.77° remained unchanged. However, the intensity of the peaks decreased significantly upon treatment with dilute HCl, indicating leaching. The magnetic adaptability also decreased, indicating a substantial reduction.

**Fig. 4:**
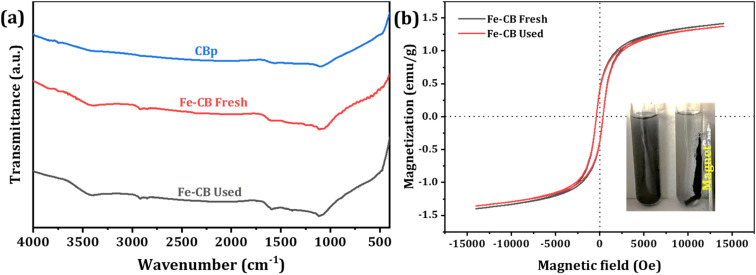
(a) FTIR spectra of CBp, fresh Fe-CB, and used Fe-CB, (b) magnetization curves of Fe-CB catalyst before and after use (the inset shows the separation of the Fe-CB catalyst by magnetic force).

FTIR spectroscopy is a useful technique for characterizing molecular vibrations and functional groups in diverse materials. The surface functional groups of the prepared Fenton-like catalysts were determined by FTIR, and the obtained data were shown in Fig. 4a. The strong absorption band at around 1635 nm results from the characteristic vibration of the C=C bond. Black carbon, composed mostly of carbon and aromatic compounds, exhibits this peak due to the presence of C=C bonds. The IR peak at 1120 nm typically results from C–O bonds in carbonyl groups, which contain a carbon atom double-bonded to an oxygen atom. Although the exact cause of this peak in black carbon is not clear, it may be related to oxygen-containing functional groups or impurities in the sample. However, the position and intensity of IR peaks may vary depending on the sample and experimental conditions. Therefore, further analysis is required to understand the composition of the black carbon sample fully. FTIR spectroscopy remains a valuable tool for studying molecular vibrations and functional groups in various materials.^44^ FTIR spectra suggest that the sample contains Fe_2_O_3_. The peak at 1061 cm^−1^ indicates the Fe–O stretching mode, and the peak at 1076 cm^−1^ may be a combination of the Fe–O stretching and bending modes. The peak at 3400 cm^−1^ is commonly caused by the stretching vibration of hydroxyl (–OH), and it is confirmed the existence of many –OH groups on the catalyst surface. Additionally, the C–H stretching mode at 2971 cm^−1^ may be due to organic contaminants or residual carbon. The bending vibration of Fe–O bonds is indicated by the peak at 410–450 cm^−1^. A weak peak around 1630 cm^−1^ is attributed to the bending mode of adsorbed water or OH groups on the surface of Fe_2_O_3_. The results are in agreement with the typical IR peaks of Fe_2_O_3_, and they offer valuable insights into the composition and surface characteristics of the sample.^45^

The magnetic properties of Fe-CB were measured using a vibrating sample magnetometer (VSM) before and after use, as shown in Fig. 4b. The fresh Fe-CB had a saturation magnetization of 1.42 emu g^−1^, which only slightly decreased to 1.38 emu g^−1^ after use. This indicates that the catalyst retained most of its magnetic properties during the degradation process. Retentivity, which refers to a material's ability to retain its magnetization after an external magnetic field is removed, was nearly unchanged before and after use, with values of −0.385 and −0.384 emu g^−1^, respectively. These results suggest that the Fe-CB material has a soft magnetic behavior that is easily reversed. Coercivity, which measures the strength of the external magnetic field required to demagnetize a material, was 365 Oe and 355 Oe for fresh and used Fe-CB, respectively. The observed non-zero coercivity values in the Fe-CB catalyst samples may be due to the presence of larger particles, while most smaller particles exhibit superparamagnetic behavior.^46^ These results indicate that the Fe-CB catalyst has low remanence and low coercivity, making it easy to separate with an external magnetic field, as shown in the inset of Fig. 4b.

**Fig. 5:**
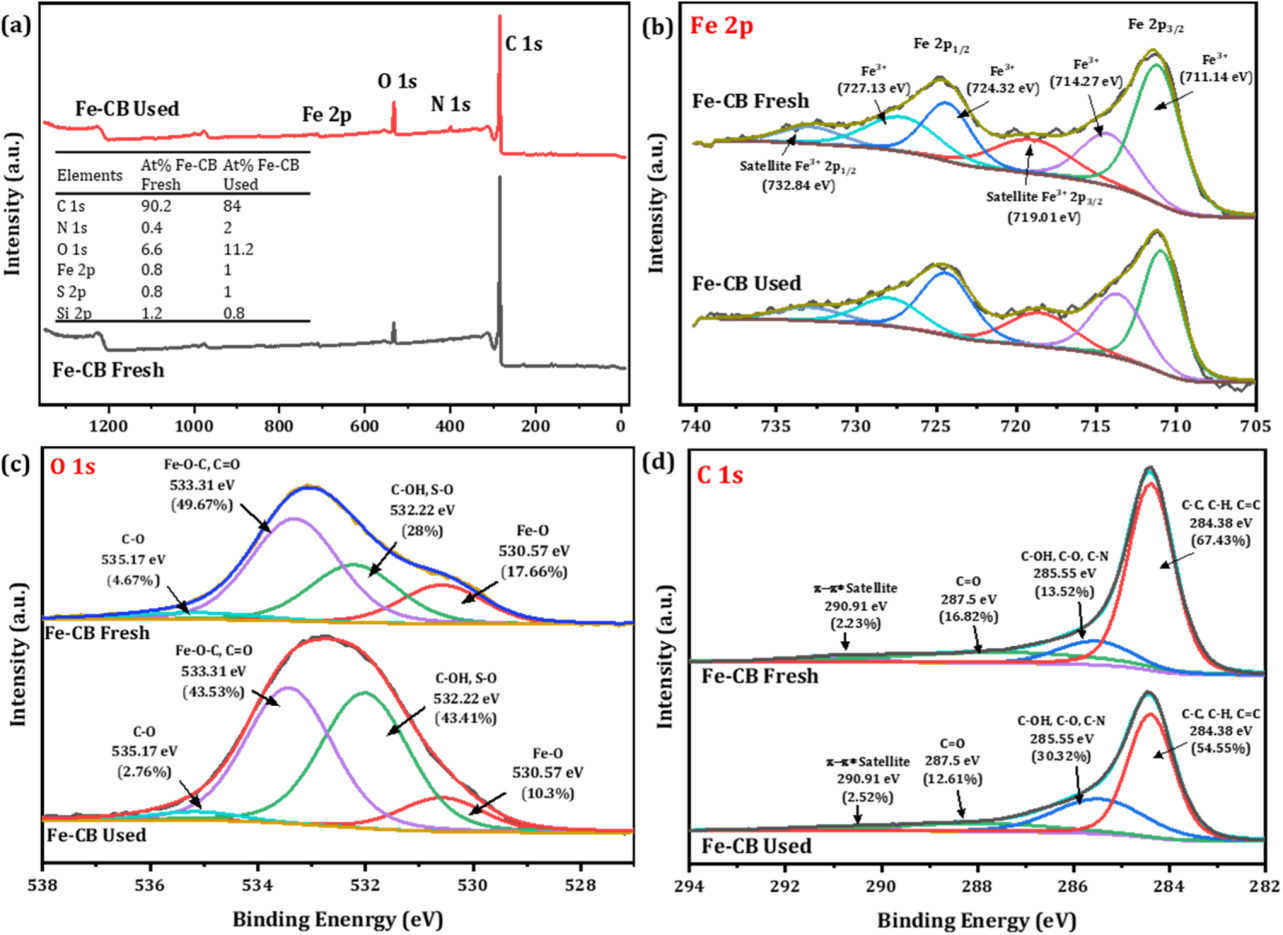
(a) Survey scan of Fe-CB before and after reaction, (the inset provides the surface compositions of the elements by atomic percentages), the high-resolution spectrum of (b) Fe 2p, (c) O 1s, and (d) C 1s of fresh and used Fe-CB catalyst.

To gain a deeper understanding of the catalyst's surface behavior during the degradation process, X-ray photoelectron spectroscopy (XPS) was used to investigate the changes in the elemental composition and oxidation states of the Fe-CB catalyst before and after the reaction. The XPS spectra for fresh Fe-CB and used Fe-CB are illustrated in Fig. 5a. As shown in Fig. 5a inset, the at% of carbon (C 1s) in fresh Fe-CB was found to be 90.2%, which decreased to 84% in Fe-CB used. On the other hand, the results show that the atomic percentage of both oxygen and sulfur increased on the catalyst surface after the reaction from 6.6% and 0.8% to 11.2% and 1.0%, respectively. This may indicate that some oxygen- and sulfur-containing functional groups were formed, such as hydroxyl, carboxyl, or sulfonic groups due to the oxidation of carbon atoms by sulfate radicals or oxygen molecules, which in turn, reduces the relative carbon content.^47^ It is also possible that persulfate molecules adsorbed on the surface of the catalyst and contributed to the increase of oxygen and sulfur at%, hence, showing the decrease in surface carbon content relative to other elements. In addition, the rise in nitrogen (N 1s) content can be observed from 0.4 at% to 2 at%. This could be explained that some nitrogen-containing species were adsorbed or incorporated into the catalyst structure, possibly from the decomposition of RhB which contained nitrogen atoms. The iron content increased slightly from 0.8% to 1% after the reaction, indicating that some iron atoms were exposed on the surface or migrated from the bulk to the surface during the reaction. The slight decrease in the silicon content from 1.2% to 0.8%, implies that some silicon atoms were lost or covered by other elements during the reaction. Silicon may originate from the carbon black support or impurities in the catalyst preparation. The deconvoluted XPS spectrum of Fe 2p (Fresh Fe-CB and Used Fe-CB) shows six peaks corresponding to Fe 2p_3/2_ and Fe 2p_1/2_ spin–orbit components and their satellites (Fig. 5b). The binding energies of the main peaks are 711.14 eV and 724.32 eV for Fe 2p_3/2_ and Fe 2p_1/2_, respectively, which are close to those reported for Fe(iii) oxide species.^48^ The satellite peaks at higher binding energies (719.01 eV and 732.84 eV) are characteristic of Fe(iii) oxides with a charge-transfer effect for Fe 2p_3/2_ and Fe 2p_1/2_, respectively.^49^ Therefore, the XPS analysis indicates that the iron on the catalyst surface is mainly in the form of Fe(iii) oxide. Fig. 5c illustrates the high-resolution XPS spectrum of O 1s which split into four peaks that correspond to different types of oxygen-containing species on the catalyst surface. The peak at 530.57 eV is assigned to Fe–O bonds in Fe(iii) oxide and the peak at 532.22 eV is attributed to C–OH and S–O bonds in hydroxyl and sulfate groups.^49^ The peak at 533.31 eV is related to Fe–O–C and C=O bonds in carboxyl and carbonyl groups and the peak at 535.17 eV is due to C–O bonds in ether and alcohol groups.^50^ The XPS analysis of O 1s revealed that the catalyst surface contains various oxygen- and sulfur-containing species. As shown in Fig. 5d, the XPS analysis of the C 1s region was deconvoluted into four peaks, revealing the presence of various carbon functionalities on the surface of the sample. The most dominant peak at 284.38 eV (67.43% area) corresponds to C–C, C–H, and C=C bonds, indicating the existence of sp^2^ or sp^3^ hybridized carbon atoms, which may form graphitic or graphene-like domains.^51,52^ The next peak at 285.55 eV, which represents 13.52% of the area, is attributed to functional groups containing oxygen and nitrogen such as C–OH, C–O–C, and C–N.^53^ The peak at 287.4 eV can be assigned to C=O bonds with a relative percentage of 16.82, which suggests the existence of carbonyl groups or carboxyl groups on the surface. The peak at 292.5 eV, which contributes 2.23% of the area, can be assigned to a π–π* satellite that arises from the shake-up process of photoemission from aromatic or conjugated structures.^53^

### 3.2. Evaluation of catalytic activity *via* degradation and mineralization of RhB

**Fig. 6:**
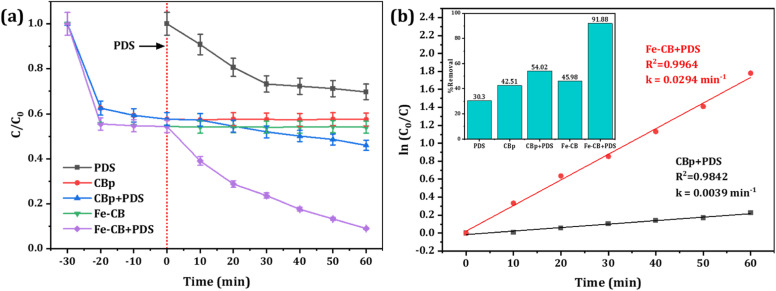
(a) Degradation of RhB under various reaction systems (PDS only, CBp, CBp + PDS, Fe-CB, Fe-CB + PDS) (b) Plots of ln(*C*_0_/*C*) *versus* reaction time of Fe-BC/PDS. Reaction condition: RhB: 20 mg L^−1^, catalyst: 1.5 g L^−1^, PDS: 10 mmol L^−1^, under neutral condition.

The study aimed to compare the efficacy of different degradation processes, including PDS without a catalyst, CBp, CBp/PDS, Fe-CB, and Fe-CB/PDS, in degrading 20 mg L^−1^ RhB in water under specific conditions. To test the removal of RhB through adsorption, a 30 minutes adsorption–desorption experiment was conducted with Fe-CB catalyst alone (without PDS) in an RhB solution under the same experimental conditions. The results, as illustrated in Fig. 6a, showed the adsorption and degradation profile of RhB in each system. Notably, the Fe-CB/PDS system in a Fenton-like system exhibited the highest performance degradation capacity, indicating the synergistic effect of the Fe-CB catalyst in the degradation processes. Following adsorption equilibrium, catalytic degradation was conducted with the addition of PDS. The addition of PDS resulted in a significant enhancement with Fe-CB, where 92% of RhB was degraded within 60 minutes of treatment. Table 1 summarizes the latest published articles that utilize Fenton-like catalysts for the degradation of RhB in aqueous solutions. As shown in the table, the results of this proposed system are comparable to those reported in the literature.

**Table 1:** Recently published article utilizing Fenton-like catalysts in the degradation of 20 mg L^−1^ RhB from aqueous solution

Catalysts	Reagent activation	Treatment time (min)	% degradation	Ref.
Fe-modified biochar	PDS at pH 5.8	30	99	54
Fe_3_O_4_ microalgae hydrochar	H_2_O_2_ at pH 4.5	60	97	55
Green iron tea nanoparticles	Persulfate at pH 3	180	99	56
MnFe_2_O_4_	H_2_O_2_ at pH 4.8	60	92	57
Fe-loaded biochar	H_2_O_2_ at pH 6.1	50	92	58
Fe_2_O_3_–Al_2_O_3_-zeolite	H_2_O_2_ at pH 7	80	100	59
Fe-CB	PDS at pH 5	60	92	This study

The degradation of RhB in both CB/PDS and Fe-CB/PDS systems followed the pseudo-first-order kinetic model, all the coefficients of determination value (*R*^2^) are greater than 0.98. The pseudo-first-order rate constant for the CBp and Fe-CB systems was calculated to be 0.0039 and 0.294 min^−1^, respectively, as depicted in Fig. 6b. The toxicity of intermediates produced from the treatment process of a treatment method can be assessed using a bioassay that analyzes the effects of degradation byproducts on organisms such as microorganisms and plants. Identification of intermediate products and TOC removal can also be used as indicators of toxicity.^60^ Degradation is the breakdown of pollutants into smaller molecules, while mineralization completely transforms pollutants into harmless inorganic compounds such as H_2_O and CO_2_. The disappearance of a pollutant does not ensure successful treatment, as decolorization alone does not guarantee wastewater quality. Additionally, degradation by-products may be more toxic than the original pollutant.^61^ Therefore, besides the degradation of initial pollutants, it is also important to follow the degree of mineralization. To further assess the complete removal of RhB intermediates, a TOC analyzer was used to measure the degree of mineralization in a 20 mg L^−1^ RhB solution. The obtained data indicates that after 60 minutes of degradation, the mineralization rate of RhB reaches 48%, highlighting the remarkable effectiveness of the process in achieving mineralization. This finding reinforces the notion that the Fe-CB/PDS system exhibits a significant mineralization effect in removing RhB intermediates. According to published reports, the effluent dye concentrations from 14 textile factories in Iraq ranged between 20 and 50 mg L^−1^.^62^ This suggests that the proposed Fe-CB/PDS system, which was effective in removing 92% of 20 mg L^−1^ RhB, could be effective for textile industrial wastewater treatment.

### 3.3. Effect of different experimental parameters on the degradation of RhB

**Fig. 7:**
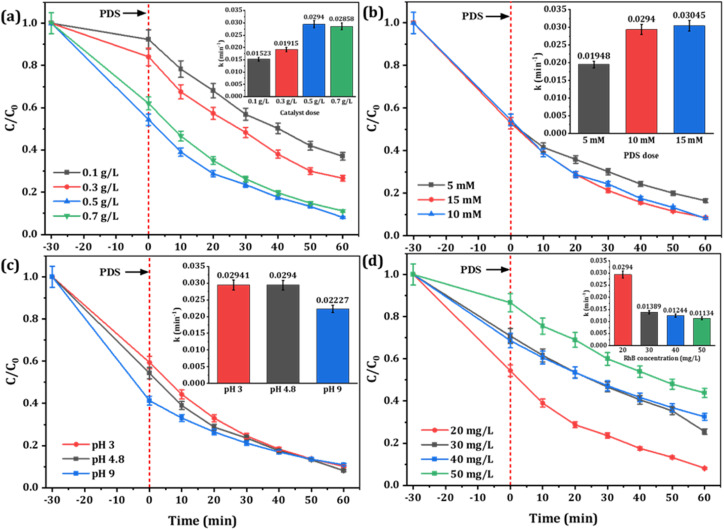
Influences of various parameters on RhB degradation: (a) catalyst dosage, (b) PDS dosage, (c) initial pH, and (d) initial RhB concentration. (Insets are the observable rate constant). Reaction condition: RhB: 20 mg L^−1^, catalyst: 0.5 g L^−1^, PDS: 10 mM, initial pH: 4.8, temperature: 24 ± 1 °C.

To systematically examine the oxidation effect of the Fe-CB/PDS system, the effects of pH, the concentration of RhB, and dosages of Fe-CB catalyst and PDS on the decomposition of RhB were investigated and the results are present in Fig. 7. The effect of catalyst dosage on adsorption and degradation of RhB by Fe-CB/PDS system was investigated by varying the catalyst dose from 0.1 to 0.7 g L^−1^, while maintaining other parameters constant (Fig. 7a). The outcomes showed that the Fe-CB catalyst had a notable adsorption capability for RhB, which increased with a rise in catalyst dosage. The adsorption of RhB on Fe-CB increased from 0.1 to 0.5 g L^−1^, reaching a maximum of 45.63%, and slightly decreased at 0.7 g L^−1^. This might be due to the aggregation of Fe-CB particles at higher doses, resulting in reduced adsorption of RhB due to a decrease in specific surface area and pore volume of Fe-CB, leading to pore filling or π–π interaction. The degradation results indicated that a higher catalyst dose led to greater degradation rates and removal efficiencies of RhB. The rate constant (*k*) increased from 0.0152 to 0.0294 min^−1^ with a rise in catalyst dosage from 0.1 to 0.5 g L^−1^, indicating that the degradation of RhB was more efficient at higher catalyst doses. The reason for this phenomenon is that the increase of catalyst dosage brings more active sites, promotes the activation of PDS to produce more sulfate radicals, thus improving the degradation efficiency of RhB. However, further increasing the catalyst dose to 0.7 g L^−1^ led to a slight decrease in *k* and percentage degradation. This could be attributed to the scavenging effect of excess catalyst on generated radicals, or the shielding effect of high turbidity caused by catalyst suspension.^63^ Another possible reason for this observation could be that the concentration of PDS remains constant. The active sites generated by the catalyst have already fully activated PDS, and further increases in catalyst dosage are unlikely to significantly enhance the degradation rate.^64^ Considering both degradation efficiency and cost considerations, a catalyst dosage of 0.5 g L^−1^ was deemed optimal for subsequent experiments in the Fe-CB/PDS system.

To determine the optimal PDS concentration, different concentrations ranging from 5 to 15 mM were tested in the Fe-CB/PDS system. The results, depicted in Fig. 7b, indicate that increasing the PDS dosage from 5 mM to 10 mM led to an improvement in the degradation rate constant from 0.0195 min^−1^ to 0.0294 min^−1^, and the removal efficiency from 83.55% to 91.88%, respectively. This enhancement can be attributed to the increased availability of sulfate radicals for oxidation.^65^ However, further increasing the PDS dosage to 15 mM did not significantly improve the removal efficiency. This may be due to the scavenging effect of excess PDS on sulfate radicals, which reduces their availability for RhB degradation (as shown in eqn (4) and (5)).^66^ Therefore, 10 mM was chosen as the optimal PDS dosage for subsequent experiments.

4 SO_4_^−^˙ + S_2_O_8_^2−^ → SO_4_^2−^ + S_2_O_8_^−^˙

5 SO_4_^−^˙ + SO_4_^−^˙ → S_2_O_4_^2−^

pH is an important factor that can greatly affect the PDS activation efficiency in heterogeneous catalysts. To investigate this effect, the Fe-CB/PDS system was tested in various pH conditions, and the outcomes are presented in Fig. 7c. At a low pH of 3, the degradation efficiency was relatively high, with a rate constant (*k*) of 0.02941 min^−1^ and a percentage removal of 89.83%. This can be explained by the fact that acidic conditions promote the production of sulfate radicals (SO_4_˙^−^), which are highly reactive and capable of efficiently oxidizing RhB molecules.^66^ At a high pH of 9, the degradation efficiency of RhB decreased slightly, with a rate constant of 0.0223 min^−1^ and a percentage removal of 89.14%. This decrease in efficiency is attributed to the formation of less reactive hydroxyl radicals through PDS decomposition, which can compete with SO_4_^˙−^ for RhB molecules.^67^ The highest degradation efficiency was observed at a slightly acidic pH of 4.8, with a rate constant of 0.0294 min^−1^ and a percentage removal of 91.88%. This pH value provides optimal conditions for the Fe-CB catalyst with PDS to effectively degrade RhB, as both SO_4_^˙−^ formation and PDS decomposition are favorable. The optimum design parameters of the proposed Fenton-like system were determined to be 10 mmol L^−1^ PDS, 0.5 g L^−1^ Fe-CB catalyst, and a reaction time of 1 hour at a wide pH range in batch mode operation.

The effect of RhB concentration on degradation efficiency was investigated by testing concentrations ranging from 20 mg L^−1^ to 50 mg L^−1^, and the results are shown in Fig. 7d. Obviously, the increase of RhB concentration is negatively correlated with the observed rate constant (*k*) and the degradation efficiency of RhB decreased gradually, with the highest *k* value obtained at the lowest RhB concentration of 20 mg L^−1^ (0.0294 min^−1^) compared to 30 mg L^−1^ (0.0139 min^−1^), 40 mg L^−1^ (0.0124 min^−1^), and 50 mg L^−1^ (0.0113 min^−1^). The highest percentage removal of RhB (91.88%) was observed at the lowest RhB concentration of 20 mg L^−1^, with decreasing removal percentages of 74.46%, 67.47%, and 56.14% at concentrations of 30 mg L^−1^, 40 mg L^−1^, and 50 mg L^−1^, respectively. The percentage of RhB adsorbed onto the catalyst surface also decreased with increasing RhB concentration, with the highest value of 45.63% observed at 20 mg L^−1^. This decreasing efficiency with increasing RhB concentration may be attributed to catalyst surface saturation, limiting the availability of active sites for further reaction. It has also been reported that the high concentration of pollutants may lead to many intermediates competing with pollutants for active free radicals. The excessive pollutants may also block the contact between catalyst and PDS, resulting in the formation of active substances cannot be timely,^68^ thus slowing down the degradation efficiency of pollutants and incomplete degradation of pollutants. Therefore, the optimal RhB concentration for efficient degradation using the Fe-CB catalyst with PDS is 20 mg L^−1^, balancing the availability of active sites for the reaction and the saturation of the catalyst surface. Moreover, it is noteworthy that RhB can be rapidly and completely degraded across all concentration gradients, which proves that Fe-CB/PDS system can be used to treat polluted wastewater with a wide range of RhB concentration and has good practical application potential.

### 3.4. The stability and reusability of the Fe-CB catalyst

**Fig. 8:**
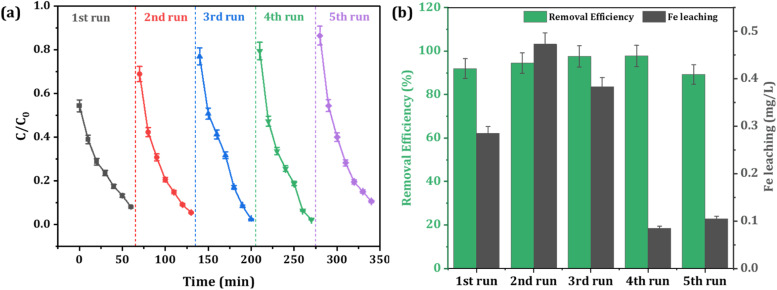
Reusability of the catalyst under optimum experimental conditions (a) degradation profile of RhB (b) leaching of iron ions after each cycle.

In practical applications, the reusability and stability of a catalyst are crucial factors that determine its value, even in the presence of notable catalytic efficiency. The recyclability and stability of the Fe-CB Fenton-like catalyst were assessed through five consecutive cycling tests. For each cycle, after the degradation process is complete, the reaction solution is poured into a filter to strain it, cleaned with deionized water, and then dried in the oven for the next cycle. The degradation rate of RhB in Fe-CB/PDS system in five cycles as shown in Fig. 8.

Surprisingly, the degradation percentage of RhB remained relatively stable, at approximately 85–90%, throughout the cycles. Although there was a slight decrease in removal efficiency by about 5%, this was likely due to the gradual loss of available adsorption sites after five cycles. The degradation rate did not exhibit a significant slowdown until the fifth cycle, highlighting the excellent stability of the catalyst and its relatively consistent high activity over time.

To evaluate the risk of secondary contamination resulting from the possible dissolution of Fe-CB catalyst, the concentrations of leached iron ions were monitored after each cycle. The iron ion leaching test after RhB degradation in the Fe-CB/PDS system was carried out by ICP-OES, the data results are shown in Fig. 8b. The results demonstrates that the concentration of Fe ions remained below 0.5 mg L^−1^ after each cycle. This concentration is well below the WHO's emission standard for Fe in drinking water, indicating that the synthesized Fe-CB catalyst is exceptionally stable and safe for use. It is worth noting that the Fe-CB catalyst can be directly reused without requiring any regeneration or activation, making it a practical and efficient option for practical applications. The synthesized Fe-CB catalyst has shown great potential for wastewater treatment, but there are still some challenges that need to be addressed before it can be used in real applications. One challenge is to study its ability to remove other organic pollutants in real wastewater and different water matrices. This would help to ensure that the catalyst is effective in a variety of conditions. Another challenge is to conduct a detailed cost assessment on a practical scale to highlight the benefits of the proposed system.

### 3.5. Scavenging experiments for identification of the dominant ROS

**Fig. 9:**
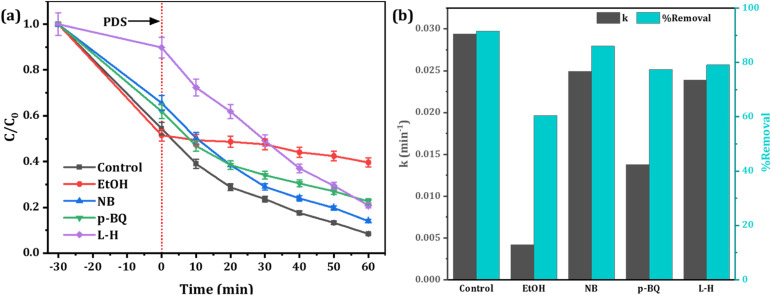
Scavenging test (a) degradation efficiency of RhB under various quenching agents (b) their corresponding rate constant.

Experimental evidence suggests that reactive oxygen species (ROS) such as hydroxyl radicals (˙OH), sulfate radicals (SO_4_˙^−^), superoxide (O_2_^−^˙) and singlet oxygen (^1^O_2_) play a crucial role in the removal of targeted pollutants in Fenton-like AOPs.^69^ Accordingly, different quenchers were used to explore the main ROS responsible for RhB degradation in the Fe-CB/PDS system. Specific scavenging reagents (EtOH, NB, *p*-BQ, and L-H) were added respectively as the scavengers for (˙OH and SO_4_˙^−^), ^˙^OH, O_2_^−^˙, and ^1^O_2_ (Fig. 9). The results obtained demonstrate that SO_4_^−^˙ was the predominant ROS accountable for the degradation of RhB, whereas O_2_^−^˙ played a secondary role in the process. These findings provide insights into the efficiency of Fe-CB/PDS Fenton-like systems in generating SO_4_^−^˙ > O_2_^−^˙ > ˙OH > ^1^O_2_ for the efficient removal of organic pollutants from contaminated water. When l-histidine is added, the degradation rate of RhB is 0.0239 min^−1^, indicating the minor role of ^1^O_2_ in the Fe-CB/PDS system.

### 3.6. Proposed mechanism of Fe-CB activating PDS in Fenton-like system

**Fig. 10:**
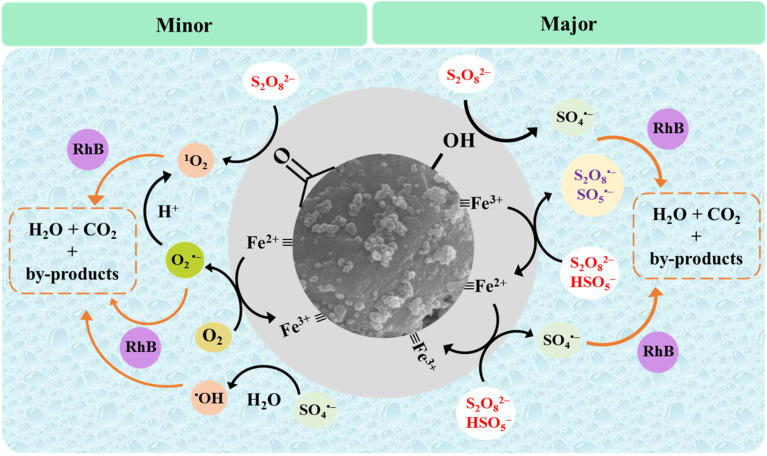
Schematic diagram of the proposed mechanism of persulfate activation in the Fe-CB/PDS system.

The possible catalytic mechanism of the generation of ROS in Fe-CB/PDS system for the removal of RhB was proposed by quenching experiment, reusability studies, characterization analysis, and a literature review (eqn (6)–(18)) (Fig. 10).^70–72^ The proposed model highlights the important role of Fe-CB as a catalyst for persulfate activation and provides valuable insights for optimizing Fe-CB/PDS systems for various applications, including environmental remediation. The reaction of persulfate anion (S_2_O_8_^2−^) with water can produce HSO_5_^−^ according to eqn (6). The mechanism of PDS activation by synthesized catalyst is mainly determined by the valence state of transition metal ions on its surface. The activation of persulfate (S_2_O_8_^2−^) in the Fe-CB/PDS system is initiated by the interaction of the persulfate anion with iron species present on the Fe-CB surface. Based on the XPS analysis, the metal ions on the catalyst surface mainly exist in the form of Fe^3+^ in the form of Fe_2_O_3_. The Fe(iii)-CB can react with S_2_O_8_^2−^ and HSO_5_^−^ to produce Fe(ii)-CB, S_2_O_8_˙^−^ and SO_5_˙^−^ (eqn (7) and (8)). The Fe(ii)-CB can, in turn, can activate S_2_O_8_˙^−^ and SO_5_˙^−^ to generate sulfate radicals (SO_4_˙^−^) *via* electron transfer as shown in eqn (9) and (10). In addition, the reusability study revealed that in the second run, the efficiency of the used catalyst increased, possibly due to the increased proportion of surface oxygen functional groups (OFGs) based on the XPS result (Fig. 10). Therefore, it can be concluded that OFGs such as carbonyl and hydroxyl groups can also play a role in the PDS activation to generate SO_4_^˙−^ (eqn (11) and (12)). Additionally, the sulfate radicals may also undergo secondary reactions to produce hydroxyl radicals (˙OH) (eqn (13)). However, the ˙OH has a minor contribution to the RhB degradation based on the quenching experiments. On the other hand, the production of superoxide and singlet oxygen can also occur in the Fe-CB/PDS system, albeit to a lesser degree. Superoxide (O_2_˙^−^) can be produced by reducing dissolved oxygen by Fe(ii)-CB with the aid of OFGs (eqn (11)). Afterward, O_2_˙^−^ can react with either hydrogen ions to form ^1^O_2_ and H_2_O_2_ (as per eqn (12)), or with ˙OH to generate ^1^O_2_ (as indicated by eqn (13)).^70^ Additionally, the activation of S_2_O_8_^2−^*via* the carbonyl groups present on the catalyst surface can also lead to the production of ^1^O_2_.^72^ The generated ROS, primarily the sulfate radical, participates in the oxidative degradation of organic pollutants. The high reactivity of the sulfate radical allows it to abstract hydrogen atoms or add to double bonds of RhB, resulting in the formation of intermediate species. These intermediate species can further undergo a series of reactions, including oxidation, reduction, and hydrolysis, ultimately leading to the formation of simpler inorganic ends products, such as CO_2_, H_2_O, and inorganic ions (eqn (18)).

6 S_2_O_8_^2−^ + H_2_O → HSO_5_^−^ + HSO_4_^−^

7 Fe(iii)-CB + S_2_O_8_^2−^ → 

<svg xmlns="http://www.w3.org/2000/svg" version="1.0" width="23.636364pt" height="16.000000pt" viewBox="0 0 23.636364 16.000000" preserveAspectRatio="xMidYMid meet"><metadata>
Created by potrace 1.16, written by Peter Selinger 2001-2019
</metadata><g transform="translate(1.000000,15.000000) scale(0.015909,-0.015909)" fill="currentColor" stroke="none"><path d="M80 600 l0 -40 600 0 600 0 0 40 0 40 -600 0 -600 0 0 -40z M80 440 l0 -40 600 0 600 0 0 40 0 40 -600 0 -600 0 0 -40z M80 280 l0 -40 600 0 600 0 0 40 0 40 -600 0 -600 0 0 -40z"/></g></svg>

Fe^2+^ + S_2_O_8_˙^−^

8 Fe(iii)-CB + HSO_5_^−^ → Fe^2+^ + SO_5_˙^−^ + H^+^

9 Fe(ii)-CB + S_2_O_8_^2−^ → ≡Fe^3+^ + SO_4_˙^−^ + SO_4_^2−^

10 Fe(ii)-CB + HSO_5_^−^ → ≡Fe^3+^ + SO_4_˙^−^ + OH^−^

11 Fe(ii)-CB + O_2_ → ≡Fe^3+^ + O_2_˙^−^

12 2O_2_˙^−^ + 2H^+^ → ^1^O_2_ + H_2_O_2_

13 O_2_˙^−^ + OH˙ → ^1^O_2_ + OH^−^

14 CB_surf._ –OOH + S_2_O_8_^2−^ → SO_4_˙^−^ + HSO_4_^−^ + CB_surf._ –OO˙

15 CB_surf._ –OH + S_2_O_8_^2−^ → SO_4_˙^−^ + HSO_4_^−^ + CB_surf._ –O˙

16 SO_5_˙^−^ → SO_4_˙^−^ + O_2_

17 SO_4_˙^−^ + H_2_O → OH˙ + SO_4_^2−^ + H^+^

18 SO_4_˙^−^/OH˙/^1^O_2_/O_2_˙^−^ + RhB → CO_2_ + H_2_O + by product

## 4. Conclusions

In this study, iron-loaded carbon black was successfully synthesized *via* chemical vapor deposition from tire wastes and used to activate PDS for the degradation of RhB as a model organic dye pollutant. The prepared catalyst exhibited excellent catalytic performance for PDS activation and efficient removal of organic dyes from water. The synthesized iron-loaded carbon black demonstrated excellent recyclability and reusability, indicating its potential for practical applications in wastewater treatment and offering a sustainable solution for the removal of organic contaminants. According to the results of quenching experiments, the degradation of RhB was mainly caused by SO_4_˙^−^ and O_2_˙^−^, while ˙OH and ^1^O_2_ played a minor role in RhB degradation. The concentration of iron ions leached into the treated solution was found to be compliant with WHO drinking water standards, indicating practical applications. This study introduces a promising method for developing resilient Fenton-like catalysts that can effectively remove organic contaminants from industrial wastewater, while also offering a new approach to waste utilization. In the synthesized Fe-CB Fenton-like catalysts, iron(iii) plays a key role in the degradation process. The Fe-CB catalyst prepared through this method is highly recyclable, easy to use, and represents a novel solution for removing organic dye pollutants from wastewater. As such, this catalyst shows significant potential for practical application.

## Author contributions

Conceptualization, Kosar H. Hama Aziz and Harez R. Ahmed; software, Kosar H. Hama Aziz, Harez R. Ahmed and Fryad S. Mustafa; validation, Kosar H. Hama Aziz, Fryad S. Mustafa and Harez R. Ahmed; writing original draft, Kosar H. Hama Aziz, Harez R. Ahmed and Fryad S. Mustafa; writing review and editing, Kosar H. Hama Aziz, Harez R. Ahmed, Fryad S. Mustafa, Nian N. M. Agha and Steven John Hinder.

## Conflicts of interest

The authors declare no conflict of interest.

## Supplementary Material
